# VT ablation based on CT imaging substrate visualization: results from a large cohort of ischemic and non-ischemic cardiomyopathy patients

**DOI:** 10.1007/s00392-023-02321-1

**Published:** 2023-12-19

**Authors:** F. Englert, F. Bahlke, N. Erhard, H. Krafft, M.-A. Popa, E. Risse, C. Lennerz, S. Lengauer, M. Telishevska, T. Reents, M. Kottmaier, C. Kolb, G. Hessling, I. Deisenhofer, F. Bourier

**Affiliations:** Department of Electrophysiology, German Heart Center Munich, Technical University of Munich (TUM), Lazarettstr. 36, 80636 Munich, Germany

**Keywords:** VT ablation, inHEART, Cardiomyopathy, Wall thinning, Late enhancement

## Abstract

**Introduction:**

The eradication of ventricular tachycardia (VT) isthmus sites constitutes the minimal procedural endpoint for VT ablation procedures. Contemporary high-resolution computed tomography (CT) imaging, in combination with computer-assisted analysis and segmentation of CT data, facilitates targeted elimination of VT isthmi. In this context, inHEART offers digitally rendered three-dimensional (3D) cardiac models which allow preoperative planning for VT ablations in ischemic and non-ischemic cardiomyopathies. To date, almost no data have been collected to compare the outcomes of VT ablations utilizing inHEART with those of traditional ablation approaches.

**Methods:**

The presented data are derived from a retrospective analysis of *n = *108 patients, with one cohort undergoing VT ablation aided by late-enhancement CT and subsequent analysis and segmentation by inHEART, while the other cohort received ablation through conventional methods like substrate mapping and activation mapping. The ablations were executed utilizing a 3D mapping system (Carto3), with the mapping generated via the CARTO® PENTARAY™ NAV catheter and subsequently merged with the inHEART model, if available.

**Results:**

Results showed more successful outcome of ablations for the inHEART group with lower VT recurrence (27% vs. 42%, *p < *0.06). Subsequent analyses revealed that patients with ischemic cardiomyopathies appeared to derive a significant benefit from inHEART-assisted VT ablation procedures, with a higher rate of successful ablation (*p = *0.05).

**Conclusion:**

Our findings indicate that inHEART-guided ablation is associated with reduced VT recurrence compared to conventional procedures. This suggests that employing advanced imaging and computational modeling in VT ablation may be valuable for VT recurrences.

**Graphical abstract:**

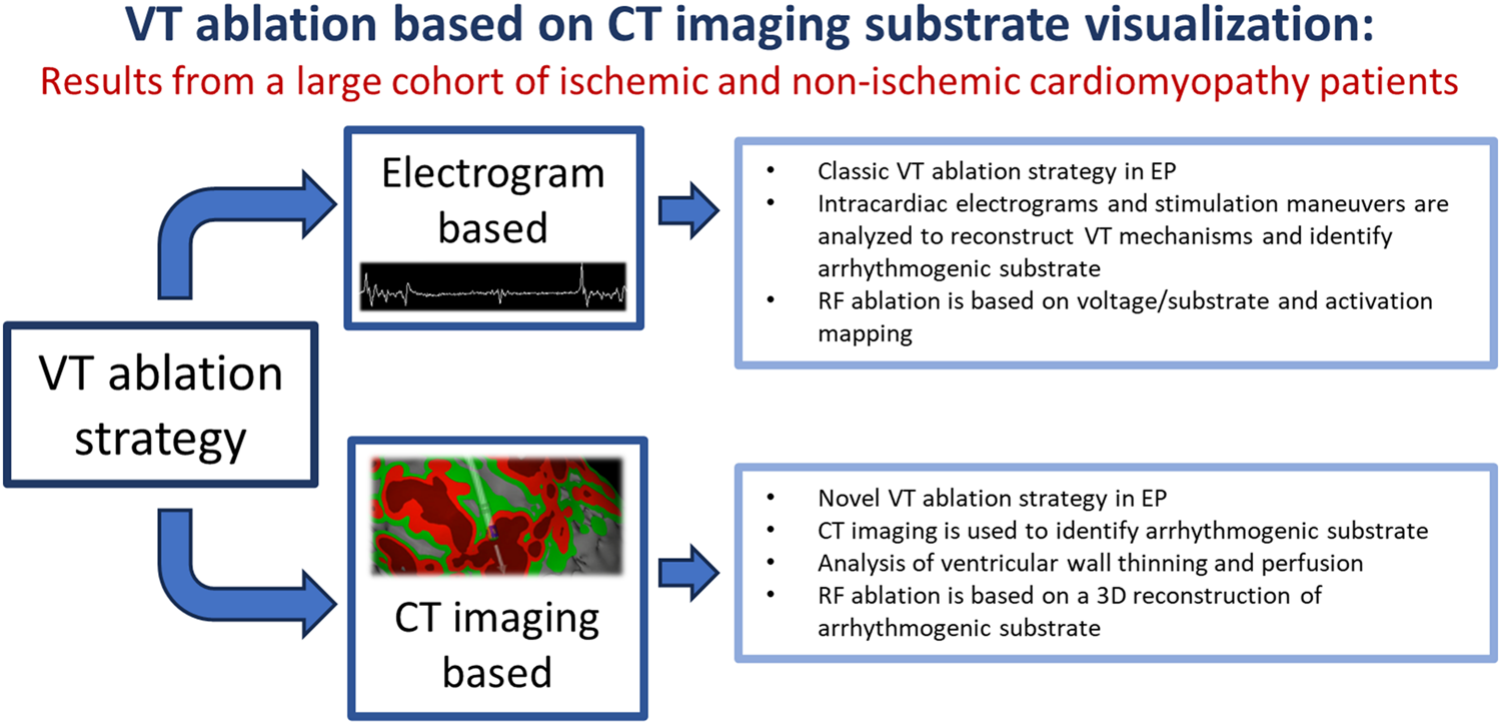

## Introduction

Ablation of ventricular tachycardia remains a clinical challenge. Since the early days of electrophysiology, this procedure has become an important procedure and remains one of the most critical interventions in the field [1]. It has been demonstrated to effectively treat life-threatening electrophysiological clinical presentations [2]. For several years, conventional stimulation maneuvers and activation mapping of sustained ventricular tachycardia (VT) were considered the preferred investigative techniques [3]. However, these methods were eventually supplanted by substrate-based methods, which are now commonly utilized, often in conjunction with activation mapping [4–7]. Regardless of the approach, all previous methods relied on the collection and analysis of intra-cardiac electrocardiograms (ECGs). In contrast, the inHEART system (IHU Liryc, Pessac, France) represents a novel approach that employs high-resolution cross-sectional imaging instead of electrical signal analysis. This new method is based on the image morphological assessment of the ventricles, in particular the anatomy of the ventricular myocardium, as well as determining the myocardial wall thickness, particularly in the case of ischemic VTs, as well as the perfusion of the myocardium.

The preliminary clinical results of the imaging-based ablation indicate promising outcomes. The objective of this study is to investigate this novel technology in a large patient cohort with various underlying cardiomyopathies.

## Methods

### Study design

**Fig. 1:**
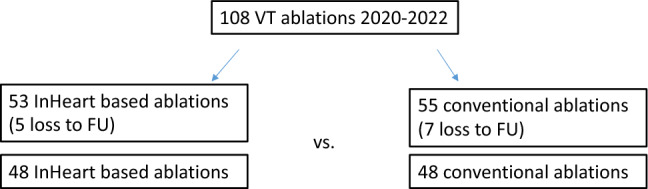
Flowchart illustrating the study design

The present study is a retrospective analysis of *n = *108 patients who underwent VT ablation at the German Heart Center Munich. The inclusion period ranged from 12/2020 to 09/2022. The inHEART group comprising *n = *53 patients got a late-enhancement computed tomography (CT) imaging and segmentation by the inHEART software platform was compared to a group *n = *55 patients who were ablated without imaging data. Due to loss to follow-up, *n = *48 patients in each group could be compared. The same technologies and catheter set-ups were used in both groups (Fig. 1).

### inHEART-based ablation

**Fig. 2:**
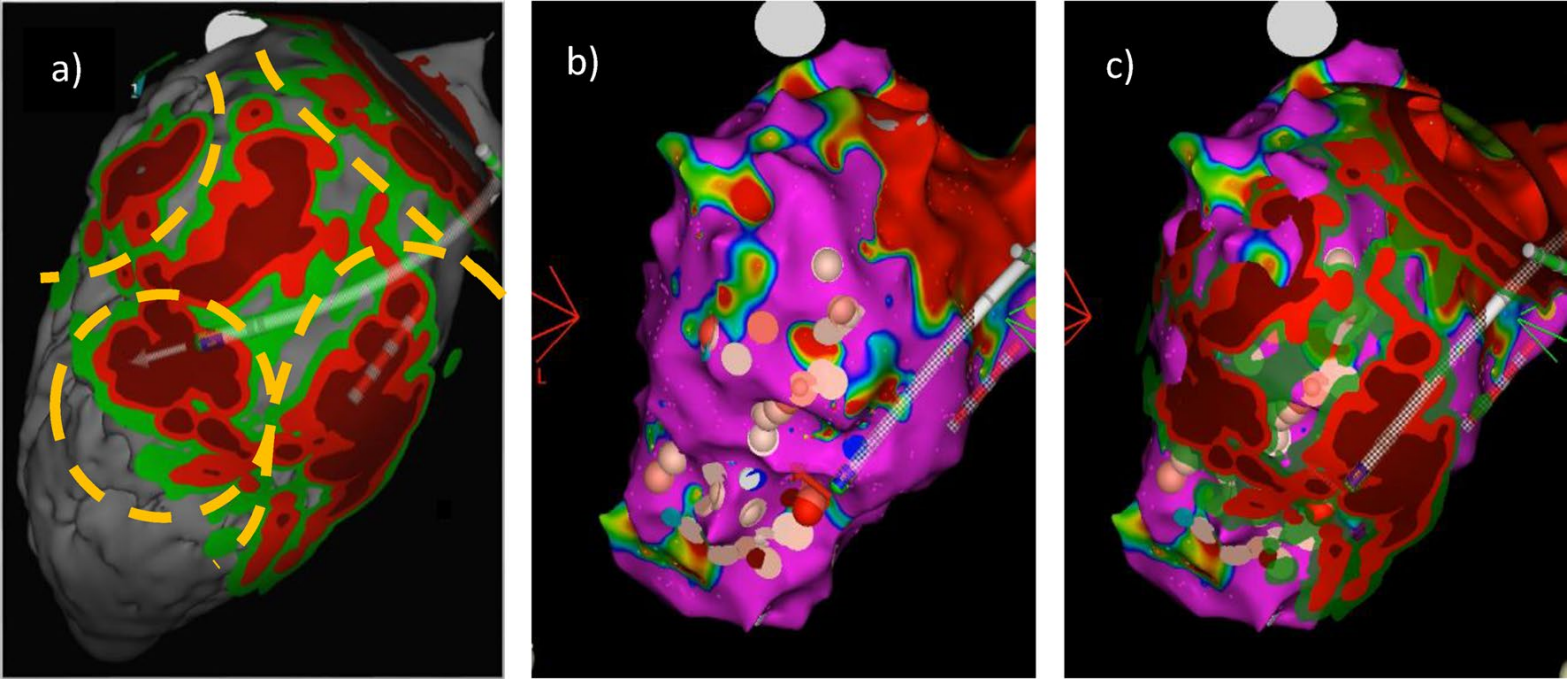
Inferior views on the left ventricle during an ep study of a dilative cardiomyopathy patient with recurrent ventricular tachycardias post myocarditis. The images A–C show corresponding views on the InHeart segmentation (**a**), the CARTO3 voltage mapping (**b**), and the merged inHeart/Voltage map (**c**). The color gradient segmentation in a) represents wall thinning and thereby the extent of left ventricular scar (gray: endocardium, green: scar as detected in late-enhancement CT, red: 2 mm, brown: 1 mm wall thickness). The dashed yellow lines represent possible VT circuits as derived from scar segmentation. The voltage map (**b**) shows healthy endocardial myocardium without relevant abnormal electrograms or arrhythmogenic substrate. C) shows the fused imaging of (**a**) and (**b**)

Prior to the procedure, the inHEART segmentation was evaluated, and potential ventricular tachycardia isthmuses were identified based on related recommendations and clinical experience [8]. The most probable isthmus of clinical VT, as well as other possibly relevant VT isthmuses, was identified when 12-lead ECG documentation was available. An anatomical map of the left ventricle (LV) was constructed using a CARTO® PENTARAY® NAV catheter and then registered with the CT segmentation via specific landmarks. Anatomical voltage mapping, as well as the reconstruction of the coronary sinus, aortic root, and LV apex for integration objectives, was conducted utilizing a 3D mapping system (Carto3, Biosense Webster, Irvine, CA). Particularly in the context of ischemic ventricular tachycardias, a remarkable correlation was observed between the voltage map and the inHeart image. The ablation procedure relied solely on the imaging data obtained through the inHEART system (Fig. 2). The equipment utilized for ablation included a THERMOCOOL SMARTTOUCH® SF catheter and the SmartAblate RF generator (Biosense Webster).

### Conventional ablation

In conventional procedures, a ventricular voltage map was acquired as a first procedural step. During voltage mapping, abnormal electrograms (local abnormal ventricular activities, fractionated and late potentials) were annotated by operator`s decision. In a second procedural step, programmed ventricular stimulation was performed to induce ventricular tachycardia. The primary ablation strategy was substrate-based ablation (abnormal electrogram elimination and loss of pace capture in scar areas). The procedural endpoint was non-inducibility of any VT in programmed stimulation after ablation.

### inHEART imaging

inHEART is a computer-based system designed to pinpoint the origins of arrhythmias. It achieves this by analyzing images obtained from various imaging modalities, such as cardiac MRI and CT scans, with a primary focus on CT imaging. CT imaging offers detailed insights into the heart's anatomy and surrounding structures. inHEART utilizes this information to construct a 3D model of the patient's heart, which can be visualized and manipulated in numerous ways to facilitate the identification of arrhythmias. It employs several CT techniques to display the heart's structural features, including contrast-enhanced CT, late enhancement, wall thinning, and perfusion CT. Contrast-enhanced CT involves the injection of a contrast agent into the bloodstream to distinguish between different heart tissues. Late enhancement imaging also uses a contrast agent, which is administered into the patient's bloodstream prior to the CT scan. The normal myocardium absorbs the contrast agent, while damaged areas, such as scar tissue, retain it for an extended period [9]. Specialized software is then used to image the contrast agent during the CT scan, differentiating normal myocardium from scar tissue. These data are utilized to generate a late-enhancement image, revealing parts of the heart where the contrast agent has been retained for a longer duration. Wall thinning is a characteristic feature of ischemic cardiomyopathy, which arises when the heart muscle's blood supply is reduced or obstructed, causing heart muscle cell death. Wall thinning CT is a technique employed to identify regions of the heart muscle that have become thinner as a result of ischemic cardiomyopathy, potentially creating VT channels [10, 11]. Perfusion CT involves the injection of a contrast agent and subsequent imaging of the heart at various time points to evaluate blood flow to the heart [12]. This method can help detect areas with diminished blood flow. Utilizing these different CT techniques, inHEART can generate comprehensive information about the heart's structure and function, which involves detailed information about myocardial scars and VT substrate.

### Follow up

Follow-up was conducted six months after the procedure via an outpatient visit, during which an interrogation of the implanted cardioverter-defibrillator (ICD) was performed. All patients had either received an ICD prior to the procedure or had one implanted shortly thereafter, with a 100% implantation rate.

### Statistical analysis

Continuous variables are expressed as mean ± standard deviation and compared by *t* tests. Categorical variables are presented as frequencies or percentages and compared by *χ*^2^ tests. Time to first VT recurrence was plotted using the Kaplan–Meier product limit method and compared by the log-rank test. Statistical tests and confidence intervals with *p < *0.05 were considered statistically significant. Statistical analysis was performed using the SPSS version 28.0 (IBM Inc., Armonk, NY).

## Results

### Patient population

A total of 108 VT patients were analyzed in this study. The inHEART group comprised 48 patients and the conventional group 48 patients as well. Patient baseline characteristics were mainly well-balanced between groups and are presented in Table 1. Mean age (62.8 ± 15.43 vs. 64.8 ± 13.91, *p = *0.72), male gender (41/48 (85%) vs. 42/48 (88%), *p = *0.38), mean EF (39.8 ± 12.40 vs. 37.46 ± 11.03, *p = *0.16), mean LVEDD (5.7 ± 0.92 vs. 5.95 ± 1.16, *p = *0.2), and diabetes (13/48 (27%) vs. 13/48 (27%), *p = *0,5) were similar between groups. The number of pre-ablations was significantly higher in the inHEART group (1.35 ± 1.73 vs. 0.81 ± 1.27, *p = *0.04). Less Ischemic VTs (12/48 (44%) vs. 28/28 (58%), *p = *0.07) and less hypertension (27/48 (56%) vs. 37/48 (77%), *p = *0.01) were found in the inHEART group.

**Table 1:** Table presenting the baseline characteristics of both the inHEART and conventional groups, including comparative statistical analysis with corresponding p values

Parameter		inHeart (*n = *48)	Conventional (*n = *48)	*p* value
Echo	LVEF *%*	39.8 ± 12.4	37.4 ± 11.0	0.16
	LVEDD mm	5.7 ± 0.9	5.9 ± 1.2	0.2
Baseline	Gender % male	88%	85%	0.38
	Age y	62.8 ± 15.4	64.8 ± 13.9	0.24
	Hypertension %	56	77	0.01
	Diabetes %	27	27	0.5
	Preablations	1.35 ± 1.7	0.81 ± 1.3	0.04
Cardiomyopathy	Ischemic %	44.0	58.3	0.16
	Dilated %	27.0	20.8	0.48
	post Myocarditis %	14.5	12.5	0.39
	Unspecified %	14.5	6.3	0.19
	ARVC %	0.0	2.1	0.32
Ablation data	Mean RF power w	47.96 ± 8.19	44.92 ± 4.05	0.13
	Mean force g	16.82 ± 5.51	13.89 ± 4.18	0.08
	RF duration min	25.3 ± 14.9	24.2 ± 16.3	0.35
	Procedure duration min	158.4 ± 71.1	151.2 ± 39.1	0.26
	Fluoroscopy duration sec	579.2 ± 464.2	505.1 ± 382.5	0.19
	Epicardial access %	8	8	0.5
Outcome	Recurrence %	27	42	0.06
Subgroup ischemic VTs	RF duration min	21.58 ± 12.73	27.54 ± 17.85	0.1
	Mean RF power w	48.81	47.60	0.39

### Procedural characteristics

The same proportion of procedures, specifically 4 out of 48 (8%), utilized epicardial access in both instances. Non-inducibility with programmed stimulation (S1/S2/S3/S4) of any VT was reached in 99% of ablation procedures. Procedure duration (158.4 ± 71.1 vs. 151.2 ± 39.1 min, *p = *0.26), radiofrequency duration (25.3 ± 14.9 vs. 24.2 ± 16.3 min, *p = *0.35), and fluoroscopy duration (579.2 ± 464.2 s vs. 505.1 ± 382.5 s, *p = *0.19), as well as mean RF power (47.96 ± 8.19 w vs 44.92 ± 4.05 w, *p = *0.13) and mean RF force (16.82 ± 5.51 g vs 13.89 ± 4.18 g, *p = *0.08) were balanced between the groups.

### Complications

The rate of pericardial effusion ≥ 10 mm was similar in the inHEART and the conventional group (1/48 (2%) vs. 1/48 (2%), *P = *0.5). All cases of pericardial effusion presented spontaneous remission in control echocardiography 48 h after the procedure and had completely resolved on follow-up visit. A single participant in the inHEART cohort required extracorporeal membrane oxygenation (ECMO) due to electromechanical decoupling. Following an extended period of intensive care, the patient was discharged without evidence of neurological impairment.

### Ablation outcomes

**Fig. 3:**
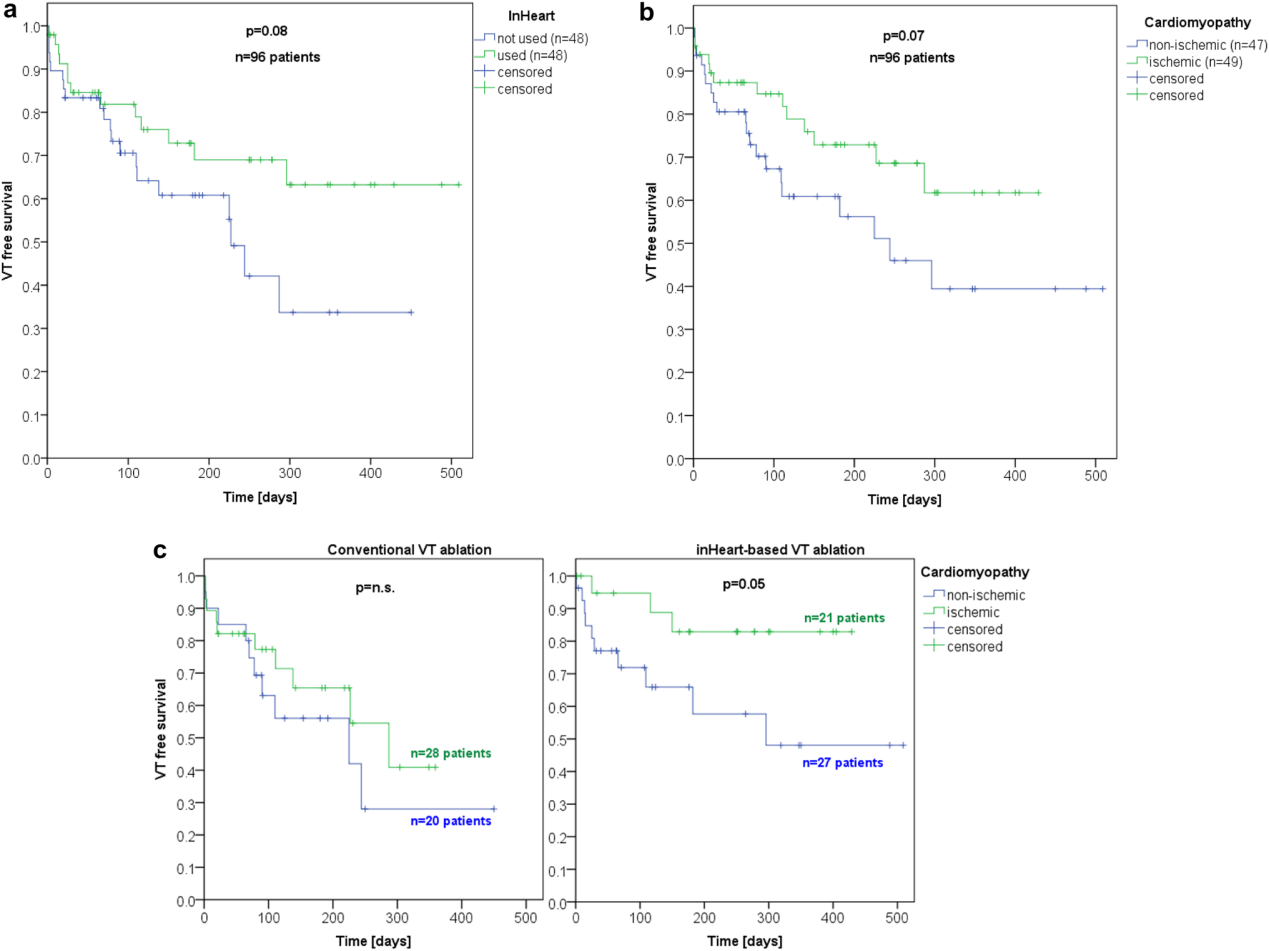
**a** Kaplan–Meier curve demonstrating the effect of inHEART-guided ablation on VT-free survival in comparison to conventional ablation strategies. **b** Kaplan–Meier curve depicting freedom from VT in patients with ischemic and non-ischemic cardiomyopathy. **c** Kaplan–Meier curve illustrating the influence of cardiomyopathy on VT-free survival, comparing outcomes between inHEART-guided ablation and conventional ablation approaches

Recurrence of VT was less observed in the inHEART group 13/48 (27%) than in the conventional ablation group 20/48 (42%) (*p = *0.06). Corresponding Kaplan–Meier curve shows similar findings (Fig. 3a). Irrespective of the treatment modality, patients diagnosed with ischemic cardiomyopathies exhibited superior outcomes in comparison to their non-ischemic counterparts (*p = *0.07) (Fig. 3b).

Subsequent analyses revealed that patients with ischemic cardiomyopathies appeared to derive a significant benefit from inHEART-assisted VT ablation procedures, with a higher rate of successful ablation (*p = *0.05) (Fig. 3c). Patients with non-ischemic cardiomyopathies who underwent inHEART-assisted VT ablation procedures had a better recurrence-free rate than without inHEART guiding (63% vs. 50%) (Fig. 3c).

## Discussion

Ventricular tachycardia is a serious arrhythmia that can lead to life-threatening complications, such as heart failure and sudden cardiac death. A promising approach for treating VTs is catheter ablation, which is based on elimination of cardiac tissue relevant to sustain the VT mechanism. However, the success rate of standard VT ablation is limited as relevant parts of the VT arrhythmia mechanism may be undetectable in conventional substrate or activation mapping.

Image-guided VT ablation is a novel approach that utilizes magnetic resonance imaging (MRI), and computed tomography, merged into 3D electro-anatomic mapping, to guide the ablation procedure [13].

### Main findings

In this study, inHeart increased the rate of successful VT ablation by 15% across all ablations performed (Fig. 3a). This improvement was found in ischemic as well in non-ischemic VT ablation procedures. In inHeart-based ablations, ischemic and non-ischemic patients achieved favorable outcomes (85.7% and 63% success rates), statistically these results were not significant. A subgroup analysis of the procedures which used inHeart showed significant better results in the ischemic group (*p = *0.05).

Upon examining the subgroup analysis of ischemic ventricular tachycardias, there appears to be no discernible difference in the intensity of ablation energy utilized. However, a noticeable extension in the duration of radiofrequency (RF) ablation is observable within the conventional group, although this deviation lacks statistical significance. This pattern could potentially signify a more focused and specific ablation strategy being facilitated by the integration of inHeart technology.

### CT imaging-guided and substrate mapping-based ablation

The CT-based ablation method features a guidance of VT ablation procedures using a non-electrogram-based approach for the first time. This approach is based on the direct visualization of the underlying VT substrate, rather than deriving intra-cardiac ECG in order to reconstruct VT mechanisms (Fig. 2) [14]. In contrast, conventional ablation methods have only been able to indirectly display structural damage through electrograms, and only to a depth of up to 1 mm on the surface [15]. In the case of a sub-endocardial, mid-myocardial, or even an epicardial substrate, these cannot be captured in intra-cardiac mapping. The CT-guided approach may allow a targeted ablation of deeper myocardial substrate, which increases the chance of successful arrhythmia elimination. In clinical practice, inHeart-guided and conventional VT ablation procedures have specific procedural similarities and differences. In both approaches, a modern 3D navigation system is used, and cardiovascular access is achieved in the same way. However, in inHeart procedures the preprocedurally available information about VT substrate localization may clarify the necessity of epicardial access. Decisive differences between inHeart and conventional approaches are encountered in VT mapping and ablation. Classic electrogram-based mapping may be abandoned in CT imaging-based procedures, using the 3D mapping system just for fast anatomic landmark collection in order to register CT-visualized substrate. In a purely inHeart-based procedure, the targets for RF ablation are selected in the CT-based VT substrate visualization and not by intra-cardiac electrogram characteristics as in conventional substrate-based ablation. However, many targeted ablation spots would correspond between both approaches and delayed, fractionated electrograms are recorded when placing the ablation electrode at positions suggested by the inHeart segmentation. The unique feature of the inHeart technology is to visualize VT substrate, which would remain concealed to purely electrogram-based mapping due to its limitations in non-endocardial substrate detection. Thus, an inHeart-based ablation may target this electrically concealed substrate, increasing procedural success rates and clinical outcome. Furthermore, the operator might achieve a more comprehensive understanding of possible VT circuits based on a complete substrate segmentation (Fig. 2). Electrogram-based mapping might underestimate the extent of myocardial scars due to above-mentioned limitations and possibly relevant parts of additional VT circuits might not undergo necessary ablation.

The forthcoming results of the ongoing prospective, randomized inEurHeart-Trial are pivotal to shaping treatment decisions for patients based on these observation [16]. Thus, we need to await these randomized trial results before considering inHeart as a clinical decision tool.

### inHeart patient cohort characteristics

A considerable number of patients had undergone previous ablations, with more patients in the inHEART group (56.3% vs 45.8%) having had prior ablation procedures. Specifically, patients who had undergone unsuccessful ablations at other medical facilities were referred to our institution as a tertiary care center were administered inHEART imaging. Notably, the inHEART group comprised a significant proportion of patients with previous unsuccessful ablations, making them particularly challenging clinical cases. However, the inHEART group demonstrated a superior performance compared to the control group.

## Limitations

This is a retrospective, non-randomized single-center study with the known inherent limitations of this study design. The significantly higher number of previously failed ablations in the inHeart group may indicate more challenging procedures in those patients and also represents a selection bias of this retrospective study. Ablation procedures expected to be more challenging, especially in non-ischemic cardiomyopathy, were significantly more often referred to the inHEART group. Despite similarities in certain baseline characteristics, factors such as hypertension were not evenly distributed between the two groups, resulting in non-homogeneous cohorts for comparison. Given the retrospective nature of this study, the available data may not be sufficient for investigating rare occurrences or long-term outcomes within the VT patient population. Additionally, loss to follow-up could potentially introduce bias or impact the generalizability of the study findings.

## Conclusion

This retrospective analysis indicates that CT imaging-guided VT ablation may increase the rate of successful ablation procedures in ischemic as well as in non-ischemic cardiomyopathy patients. Whereas conventional procedures rely on intra-cardiac electrogram analysis, inHeart-guided VT ablation is based on anatomic and functional imaging. The novel technique thus has the potential to visualize VT substrate beyond conventional electrogram-based mapping.

## Data Availability

The data are available from the authors upon reasonable request.
